# Simple against advanced imaging for the selection of stroke therapy in the extended window (VESTA study)

**DOI:** 10.1093/esj/aakag068

**Published:** 2026-06-17

**Authors:** Adrián Valls-Carbó, Mònica Millán, Carlos Castaño, Natalia Pérez De La Ossa, Pere Cardona, Alan Flores, Carles Biarnes, Martí Boix, Xabier Urra, Mikel Terceño, Marta Olivé-Gadea, Pol Camps Renom, Ana Rodríguez-Campello, Francisco F Purroy, Bárbara Yugueros, David Cánovas, Isabel Rodriguez Caamaño, Brigitte Beltrán-Mármol, Sebastià Remollo, Marta De Dios, Saima Bashir, Lucia Aja, Laura Ludovica Gramegna, Miriam Pastrana, María Hernández-Pérez

**Affiliations:** Department of Neurosciences, Germans Trias i Pujol University Hospital, Germans Trias i Pujol Research Institute, Universitat Autònoma de Barcelona, Badalona, Spain; Department of Neurosciences, Germans Trias i Pujol University Hospital, Germans Trias i Pujol Research Institute, Universitat Autònoma de Barcelona, Badalona, Spain; Department of Neurosciences, Germans Trias i Pujol University Hospital, Germans Trias i Pujol Research Institute, Universitat Autònoma de Barcelona, Badalona, Spain; Department of Neurosciences, Germans Trias i Pujol University Hospital, Germans Trias i Pujol Research Institute, Universitat Autònoma de Barcelona, Badalona, Spain; Department of Neurology, Hospital Universitari Bellvitge, Barcelona, Spain; Department of Neurology, Hospital Joan XXIII, Tarragona, Spain; Institut d'Investigació Biomèdica de Girona, Hospital Doctor Josep Trueta de Girona, Girona, Spain; Department of Neurosciences, Germans Trias i Pujol University Hospital, Germans Trias i Pujol Research Institute, Universitat Autònoma de Barcelona, Badalona, Spain; Department of Neurology, Hospital Clínic de Barcelona and Institut d'Investigacions Biomèdiques August Pi i Sunyer (IDIBAPS), Barcelona, Spain; Neurology Department, Hospital Doctor Josep Trueta de Girona, Girona, Spain; Stroke Unit, Department of Neurology, Hospital Vall d'Hebron, Barcelona, Spain; Stroke Unit, Department of Neurology, Hospital de Sant Pau i la Santa Creu, Barcelona, Spain; Stroke Unit, Department of Neurology, Hospital del Mar, Barcelona, Spain; Department of Neurology, Hospital Arnau de Vilanova, Lleida, Spain; Department of Neurosciences, Germans Trias i Pujol University Hospital, Germans Trias i Pujol Research Institute, Universitat Autònoma de Barcelona, Badalona, Spain; Department of Neurology, Hospital Parc Taulí, Sabadell, Barcelona, Spain; Radiology, Germans Trias i Pujol University Hospital, Badalona, Spain; Radiology, Hospital del Mar, Barcelona, Spain; Radiology, Germans Trias i Pujol University Hospital, Badalona, Spain; Radiology, Hospital Vall d'Hebron, Barcelona, Spain; Neurology, Hospital Doctor Josep Trueta de Girona, Girona, Spain; Radiology, Hospital Vall d'Hebron, Barcelona, Spain; Radiology, Hospital Vall d'Hebron, Barcelona, Spain; Department of Neurosciences, Germans Trias i Pujol University Hospital, Germans Trias i Pujol Research Institute, Universitat Autònoma de Barcelona, Badalona, Spain; Department of Neurosciences, Germans Trias i Pujol University Hospital, Germans Trias i Pujol Research Institute, Universitat Autònoma de Barcelona, Badalona, Spain

**Keywords:** CTA, CTP, endovascular-treatment, ischaemic-stroke, late-window

## Abstract

**Introduction:**

The optimal imaging modality for selecting stroke patients for revascularisation in the extended window remains uncertain. The VESTA study compared simple (non-contrast CT + CTA) vs advanced imaging (including perfusion) in the extended window in terms of clinical outcomes, mortality and safety.

**Patients and methods:**

This multicentre cohort study included 1262 stroke patients (last seen well 6–24 h, NIHSS ≥ 6) from the Catalan Stroke Registry (2019-2021). A central core lab re-evaluated images, and blinded investigators assessed 90-day functional outcomes. Inverse probability weighting (IPW) and multivariable methods were applied.

**Results:**

Median age was 76 years, NIHSS 12 and 48% were women. Simple imaging was used in 44% (*n* = 550), advanced in 56% (*n* = 712). Simple imaging had higher rates of no arterial occlusion (49% vs 37%, *P* = .006) and slightly lower endovascular treatment rates (36% vs 40%, *P* = .117). Time metrics were similar. In the IPW analysis, (advanced imaging as reference), simple imaging showed numerically worse point estimates across all outcomes, although most differences did not reach statistical significance: a worse mRS shift (adjusted odds ratio [aOR] 1.17 [95% CI, 0.96–1.43]; *P* = .11), a lower good functional outcome (mRS 0–2; aOR 0.83 [0.66–1.06]; *P* = .13), a higher mortality (aOR 1.20 [0.91–1.58]; *P* = .20), more frequent sICH (aOR 1.25 [0.61, 2.57]; *P* = .55) and a higher risk of any ICH (aOR 1.57 [1.00–2.47]; *P* = .05).

**Discussion:**

In moderate-to-severe stroke (NIHSS ≥ 6) within 6–24 h, simple imaging did not show a statistically significant difference vs advanced imaging for guiding stroke treatment. However, advanced imaging may improve patient selection for reperfusion and reduce haemorrhagic risk.

**Trial Registration Information:**

This study was registered at ClinicalTrials.gov under NCT05299034.

## Graphical abstract

**Graphical Abstract:**
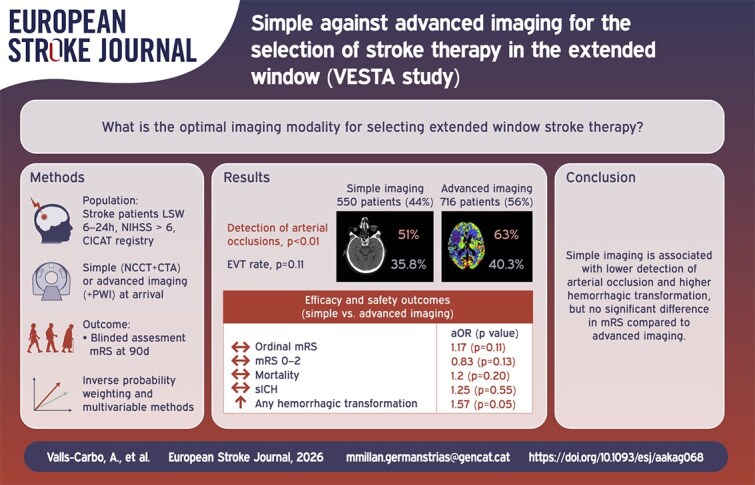

## Introduction

Current American and European stroke guidelines state that patients presenting 6–24 h after stroke onset should meet the perfusion criteria used in the DAWN or DEFUSE3 trials to receive EVT.^1–4^

Advanced imaging provides unique information about the brain’s state, helps identify arterial occlusion, and could allow better personalisation of stroke therapy. The drawbacks of this technique are higher cost, the need for post-processing methods with costly software beyond the reach of some centres, and the time required for acquisition and processing, which can lead to delayed treatments. Over the last few years, a few trials have shown that EVT is safe and effective in patients with large core selected using simple imaging. Nevertheless, these trials do not directly compare simple vs advanced paradigm approaches. Thus, there is interest in understanding whether a simpler imaging paradigm could be as good as the current advanced imaging paradigm for selecting patients for EVT in the extended window.

Several real-world evidence studies have compared simple against advanced imaging for EVT selection.^5–7^ However, these studies only included patients who underwent EVT, providing no data on those who were not treated with thrombectomy or their outcomes. This is a significant limitation, as the question of the optimal imaging modality affects all patients presenting with suspected stroke who meet clinical criteria for EVT. In such cases, imaging should guide the best therapeutic decisions—and these decisions cannot be fully understood by analysing only the subset of patients who received EVT.

In the present study, we used a target trial emulation framework to emulate a randomised experiment.^8^ We aimed to evaluate the impact of simple against advanced imaging paradigms on clinical decision-making, stroke prognosis and safety outcomes in patients with suspected stroke beyond 6 h from onset and a high likelihood of EVT.

## Patients and methods

For this retrospective study, we included consecutive patients recruited in the Catalan Stroke Registry (CICAT) between January 2019 and December 2021. Catalan Stroke Registry is a government-mandated prospective stroke registry involving all the Catalan stroke centres. Further details about the registry are available here (https://aquas.gencat.cat/ca/fem/intelligencia-analitica/registre-cicat/index.html#googtrans(ca|en). We used a target trial emulation framework, so we applied inclusion and exclusion criteria for participation in a hypothetical randomised experiment (Table S1). The study adhered to the Strobe guidelines for observational studies.

The inclusion criteria were:

1. Acute stroke with symptoms of the anterior circulation
2. NIHSS Scale ≥ 6 (moderate–severe stroke)
3. ≥18 years, with no superior age limit
4. 6–24 h from symptom onset or last time seen well
5. Admission in ESO-certified stroke centres with both neuroimaging modalities available directly or after transfer from primary or telestroke stroke centres

The exclusion criteria were:

1. Pre-stroke mRS score > 2
2. Haemorrhage at non-contrast CT (NCCT)

Our selection criteria differed from the hypothetical randomised trial criteria in the following ways: (1) we only included patients with acute stroke and clinical symptoms of the anterior circulation; we could not include all stroke suspicions (including mimics) because the registry does not collect detailed data of mimics; (2) we included patients admitted to centres in which both simple and advanced imaging are available, but in contrast to a trial, we do not know if both neuroimaging modalities were available at the same time, nor do we know the reason why one imaging modality was preferred over the other; (3) in a randomised trial, allergy to iodinated contrast should be an exclusion criterion, but we do not have information about this condition in our registry and cannot therefore reflect this information in the flow chart. As patients and families are routinely asked for previous allergies at admission, we believe that no patients with known allergy were exposed to iodinated contrast.

Local neurologists recorded demographic variables, cardiovascular risk factors, stroke severity at admission, relevant time metrics (onset to arrival, onset to imaging, onset to treatment in case of receiving reperfusion therapy) and treatment (none, intravenous thrombolysis and/or EVT). In the case of EVT, they also registered time from onset to puncture, mTICI at the end of the procedure and sICH at 24 h according to SITS-MOST criteria.^9^ Imaging selection criteria for mechanical thrombectomy differed slightly among centres (list of centres and detailed EVT criteria for each centre in Table S2). In general, patients received EVT if they had a large anterior occlusion (intracranial internal carotid artery or MCA in segments M1 or proximal M2), an ASPECTS score ≥ 6 and absence of clear hypodensity on the NCCT. When CTP was available, a predicted ischaemic core at baseline (defined as relative cerebral blood flow < 30%) < 70 mL and the existence of mismatch were commonly required.

The study was reviewed and approved by the Ethics Committee for Research at the Germans Trias University Hospital, and waiver of consent was granted (PI-22-043).

### Imaging evaluation

All images were stored in a regional repository and pseudonymised. A neuroimaging core lab composed of 7 neuroradiologists and 3 stroke neurologists from comprehensive stroke centres with expertise in neuroimaging reviewed all the images and registered the type of imaging, quality of the images, presence of ischaemic lesion (established infarct, early ischaemic signs, no ischaemic signs), Alberta Stroke Program CT score (ASPECTS), site of the occlusion and collateral grade.^10^ Image quality was assessed using a 5-point Likert scale reflecting the global diagnostic usability of the study. Quality was rated as: (1) non-diagnostic, (2) diagnostic but limited, (3) good quality, (4) very good quality or (5) excellent quality. This assessment was intended to capture the overall adequacy of the imaging study for diagnostic interpretation, including factors such as motion artefacts, contrast opacification, signal-to-noise ratio and the clarity required to reliably assess the imaging variables previously described. For CTP, central evaluators had access to the maps previously processed by each centre (predominantly using Rapid, but also Mistar, Syngo.via, IntelliSpace Portal and Brainomix). The pseudonymised images were evenly distributed among the members of the core lab, and a private access to the image repository was created for each of them, allowing access only to their assigned imaging batch. The evaluators reviewed each study individually and had access to all the sequences of the baseline imaging study of each patient. However, they were blinded to the rest of the clinical and radiological information. To assess inter-rater reliability of the central imaging variables, a random subset of 20 baseline imaging studies was evaluated by each member of the core lab. Agreement was quantified with Fleiss’ κ (Landis & Koch categories) and, for ASPECTS, with the intra-class correlation coefficient (ICC, 2-way model, absolute agreement). Inter-rater reliability was almost perfect for proximal arterial occlusion (κ = 0.84), moderate for the detection of distal (M2/M3) occlusion (κ = 0.53) and for ASPECTS (ICC = 0.45; 95% CI, 0.26–0.66). Data were collected and managed using RED-Cap electronic data tools. A complete list of the members of the imaging core lab and their expertise is available in the Supplementary Material.

### Outcome variables

Our efficacy outcomes were functional disability explored as the full range of the mRS at 3 months (as an ordinal variable) and good clinical outcome defined as a score of 0–2 in the mRS at 3 months. Functional status was evaluated centrally at 3 months by nurses certified in the mRS who were unaware of the type of imaging received by the patient.

Safety outcomes were sICH at 24 h and mortality at 90 days. Symptomatic ICH was only registered in patients receiving reperfusion treatment.

### Statistical analysis

Descriptive statistics were used to summarise the variables. Continuous variables were reported as mean and SD or median and IQR where appropriate. Categorical variables were presented as counts and percentages (*n*, %). For group comparisons, we applied the Mann–Whitney *U* test for non-normally distributed continuous variables, the Student’s *t-*test for normally distributed variables, and the chi-square test for categorical variables, as appropriate.

To adjust for confounders, inverse probability weighting (IPW) was applied using average treatment effect (ATE) formula. Under this target trial emulation framework,^11^ the exposure was defined as a compound imaging-guided strategy (simple: NCCT + CTA vs advanced: +CTP) and the estimand as its average treatment effect on 90-day mRS. The propensity score was restricted to pre-imaging variables; post-imaging information on the causal pathway (notably site of occlusion) was excluded to avoid blocking part of the effect. According to the ATE, we estimated the probability of assignment (PS) to the simple imaging protocol conditional on the following: age, sex, prior mRS score, NIHSS at baseline, time to hospital arrival (categorised as 6–10.5, 10.5–15, 15–24 h, unknown), ASPECTS (including a category for the missing values [*n* = 5]) and presence of established infarct. For the pre-specified sensitivity analyses, the PS was (a) re-estimated additionally including the baseline site of arterial occlusion as a covariate, and (b) recomputed replacing time-to-hospital-arrival tertiles with quartiles. Both sensitivity analyses yielded materially unchanged results (see “Results”). According to the ATE formula, the simple imaging group received weights of [1/PS], and the advanced imaging group received weights of 1/(1 − PS). We stabilised the weights with the marginal treatment probabilities and trimmed extreme values at the 1st and 99th percentiles. Covariate balance after weighting was verified with Love plots; all standardised mean differences were < 10% (Figure S1).

All outcome models were fitted in a survey-weighted framework that treats the IPW weights as sampling weights and uses centre of admission as the primary sampling unit. Variances and confidence intervals were obtained by Taylor linearisation, which is robust to within-centre correlation.

- Ordinal outcome (90-day mRS shift)—analysed with a weighted proportional-odds logistic model (svyolr function from survey R package, cumulative log-odds link).
- Binary outcomes (independence at 90 days, survival, any symptomatic haemorrhage)—analysed with weighted logistic regression (svyglm function from survey R package, logit link, quasibinomial family).

Results are reported as weighted adjusted odds ratios (ORs) with 95% CI; OR < 1 favours simple imaging, OR > 1 favours advanced imaging. Sensitivity analyses using trimmed ATE weights and overlap weights produced materially similar estimates (data shown upon request).

We performed subgroup analysis for the following patient groups: (1) treated with EVT; (2) not treated with EVT; (3) proximal occlusion (defined as M1 segment of the MCA or acute carotid occlusion); (4) distal occlusion (defined as M2 or more distal and anterior cerebral artery occlusion); (5) arriving directly at a comprehensive stroke centre (not transferred); (6) transferred from a primary stroke centre.

The statistical analysis was conducted using R software (version 4.4.0) and RStudio (version 2023.8.0.53). The compareGroups, WeightIt and Cobalt packages were used for descriptive and weighting analyses, while the modelsummary and broom packages facilitated the regression analysis and presentation of results. The *P*-values were 2-sided, without adjustments for multiple testing, and *P* < .05 was considered statistically significant.

## Results

### Description of the sample

**Figure 1:**
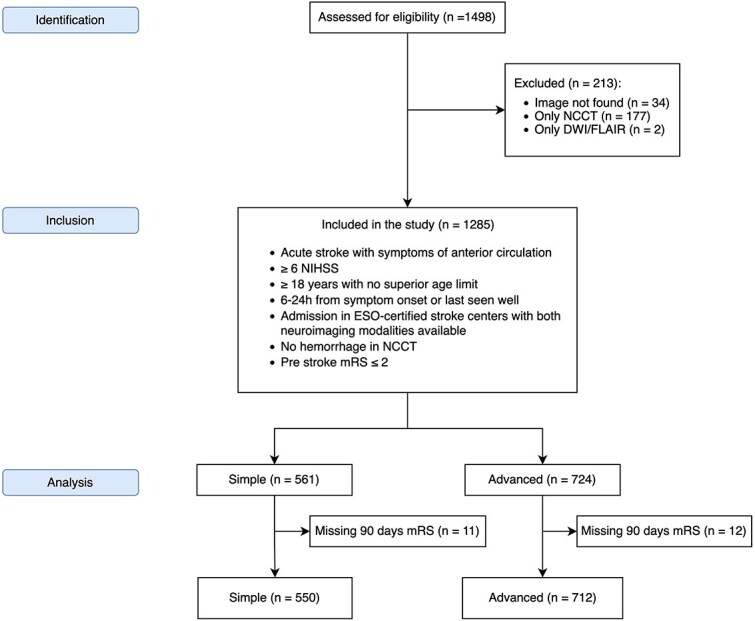
Flow chart documenting included and excluded patients and kind of neuroimaging acquired.

From January 2019 to December 2021, 1498 patients fulfilling the VESTA inclusion criteria were identified in the CICAT registry. After excluding patients in which the imaging was not available (*n* = 34), those without an angiographical imaging at admission (*n* = 179) and those lost to follow-up for the 90-day mRS assessment (*n* = 23), 1262 patients were suitable for the analysis, of which 550 (44%) received simple imaging while 712 (56%) received advanced imaging. Figure 1 represents the patient inclusion flowchart and supplementary table 3 (Table S3) shows the distribution of imaging modality across centers.

Table 1 shows the baseline characteristics and treatment variables; Table 2 illustrates the radiological features of the whole cohort according to imaging modality. Both imaging groups were similar in age, sex, cardiovascular risk factors, previous mRS and time from onset to arrival. Imaging modalities were not equally distributed among centres (Table S3). Patients receiving simple imaging had lower stroke severity (median NIHSS 11 [IQR 8;18] vs 12 [IQR 8;19], *P* = .028) and shorter onset to imaging time (624 [407;820] vs 645 [449;870] min, *P* = .037) than patients receiving advanced imaging. Simple images were less frequently rated as very good or excellent quality than advanced images (*P* < .001). The median ASPECTS score was 9 in both groups, and we found no differences between the 2 groups in leptomeningeal collaterals. Established infarcts were more frequent in patients in the simple imaging group than patients in the advanced group, though the difference was not statistically significant (21.6% vs 17.4%, *P* = .07). Simple imaging patients had a lower rate of distal occlusions (21.1% vs 24.0% *P* < .001), and a higher rate of absence of arterial occlusion (44.9% vs 37.2%, *P* = .006) when compared with the advanced imaging group. In addition, patients in the simple imaging group were slightly less frequently treated with EVT (35.8% vs 40.3%, *P* = .117). Among patients treated with EVT, the door-to-groyne time did not differ between both groups (median time: 73 vs 80 min; *P* = .176 for the simple vs advanced imaging groups).

**Table 1:** Baseline characteristics, and selected treatment in patients undergoing simple against advance imaging in the whole cohort.

	All *n* = 1262	Simple *n* = 550	Advanced *n* = 712	*P* value
**Baseline characteristics**				
**Age**	76 [64;83]	76,0 [63;83]	76 [66;83]	.466
**Sex (woman)**	608 (48%)	261 (48%)	347 (49%)	.693
**Hypertension**	413 (59%)	183 (61%)	230 (57%)	.317
**Dislipidaemia**	294 (42%)	122 (41%)	172 (43%)	.660
**Diabetes**	135 (19%)	57 (19%)	78 (19%)	.992
**Atrial fibrillation**	131 (19%)	62 (21%)	69 (17%)	.268
**mRS at baseline**				.159
** *0***	761 (60%)	348 (63%)	413 (58%)	
** *1***	274 (22%)	112 (20%)	162 (23%)	
** *2***	227 (18%)	90 (16%)	137 (19%)	
**NIHSS at admission**	12 [8;18]	11 [8;18]	12 [8;19]	.028
**Kind of facility of arrival**				.987
** *CSC***	999 (79%)	436 (79%)	563 (79%)	
** *Transferred to CSC***	263 (21%)	114 (21%)	149 (21%)	
**Known onset**	167 (12.9%)	90 (12.4%)	77 (13.7%)	.548
**Onset to arrival (min)**	608 [400;816]	599 [393;792]	612 [420;836]	.148
**Onset to puncture (min)**	650 [450;864]	660 [455;846]	645 [444;873]	.973
**Door to puncture (min)**	77.0 [51.0;110]	73.0 [47.5;110]	80.0 [55.0;107]	.176
**Treatment characteristics**				
Reperfusion treatment				
** *None***	716 (56.7%)	323 (58.7%)	393 (55.2%)	.231
** *Primary EVT***	459 (36.4%)	185 (33.6%)	274 (38.5%)	.086
** *IVT* alone**	62 (4.9%)	30 (5.5%)	32 (4.5%)	.515
** *EVT* + *IVT***	25 (2.0%)	12 (2.2%)	13 (1.8%)	.805
**Final mTICI**				.962
** *mTICI 0***	40 (8%)	17 (9%)	23 (8%)	
** *mTICI 1***	13 (3%)	6 (3%)	7 (2%)	
** *mTICI 2a***	21 (4%)	8 (4%)	13 (5%)	
** *mTICI 2b***	118 (24%)	48 (24%)	70 (24%)	
** *mTICI 2c***	67 (14%)	24 (12%)	43 (15%)	
** *mTICI 3***	218 (45%)	92 (47%)	126 (44%)	
** *mTICI unknown***	7 (1%)	2 81%)	5 (2%)	
**Symptomatic ICH**	32 (6%)	15 (7%)	17 (5%)	.658

Abbreviations: CSC, comprehensive stroke centre; IVT, intravenous treatment.

Mean ± SD, median [25th–75th percentile] or *n* (%).

**Table 2:** Radiological characteristics.

	All *n* = 1262	Simple *n* = 550	Advanced *n* = 712	*P* overall
Imaging quality				<.001
** *No diagnostic***	10 (0.7%)	7 (1.27%)	3 (0.42%)	
** *Diagnostic***	126 (9.9%)	69 (12.5%)	57 (8.01%)	
** *Good quality***	533 (42.2%)	292(53.1%)	241 (33.8%)	
** *Very good quality***	442 (35.0%)	151 (27.5%)	291 (40.9%)	
** *Excellent quality***	151 (11.96%)	31(5.64%)	120 (19.6%)	
**ASPECTS**	9 [7;10]	9 [7; 10]	9 [8;10]	.660
Lesion evolution at arrival				
** *No ischaemic signs***	539 (42.7%)	235 (42.7)	304 (42.7%)	1
** *Established infarct***	243 (19.2%)	119 (21.6%)	124 (17.4%)	.07
*Acute ischaemia*	498 (39.5%)	200 (36.4%)	298 (41.8%)	.06
Site of the occlusion				
*No occlusion*	512 (40.6%)	247 (44.9%)	265 (37.2%)	.006
*Isolated extracranial ICA*	20 (1.58%)	10 (1.82%)	10 (1.40%)	.721
*Tandem*	57 (4.52%)	19 (3.45%)	38 (5.34%)	.144
*Proximal occlusion*	374 (29.6%)	149 (27.1%)	225 (31.6%)	.093
*Distal occlusion*	287 (22.7%)	116 (21.1%)	171 (24.0%)	<.001
*Posterior occlusion*	12 (0.95%)	9 (1.64%)	3 (0.42%)	.056
Collateral grade				
** *Not evaluable***	71 (9.1%)	23 (7.59%)	48 (10.2%)	.865
** *No collaterals***	46 (5.9%)	17 (5.61%)	29 (6.18%)	
** *< 50% collaterals***	196(25.38%)	78 (25.7%)	118 (25.2%)	
** *> 50% collaterals***	252 (32.64%)	101(33.3%)	151 (32.2%)	
** *100% collaterals***	207 (26.78%)	84 (27.75%)	123 (26.18%)	

Abbreviations: ASPECTS, Alberta Stroke Program CT score; ICA, internal carotid artery.

After IPW, both imaging groups were well adjusted with standard mean differences < 0.1 for every variable (Figure S1).

### Efficacy and safety outcomes

**Figure 2:**
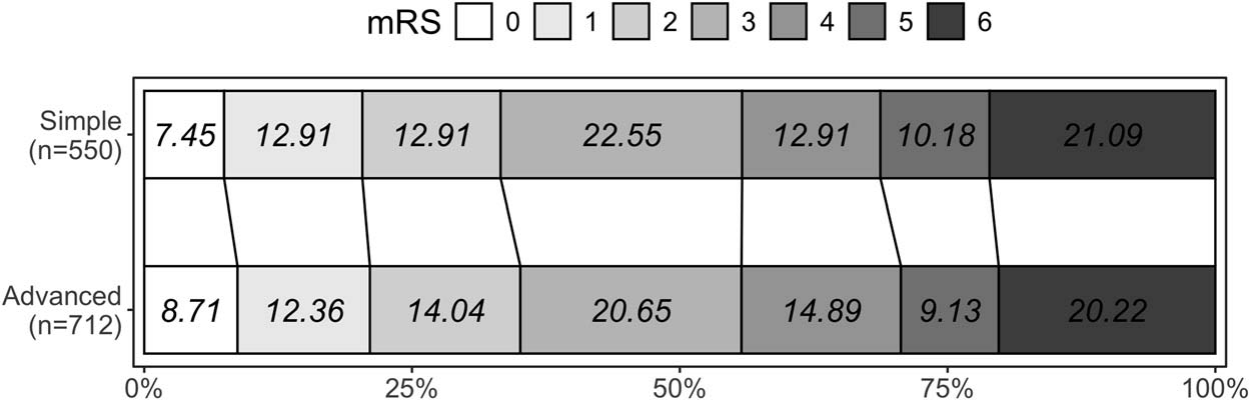
Grotta bars showing clinical outcome at 90 days in the complete cohort according to the kind of neuroimaging acquired.

When evaluating the whole cohort, the median mRS at 90 days (3 [2–5] and 3 [2–5], *P* = .582) and the rate of patients with functional independence (33.3% vs 35.1%, *P* = .533) at 3 months was similar between the simple and advanced imaging groups (Figure 2). In the subgroup of patients receiving EVT, 31.4% achieved functional independence without differences between both imaging modalities (29.4% vs 32.7%, OR 0.86 [0.58, 1.27], *P* = .44] in the simple and advanced imaging groups, respectively).

After IPW and adjustment, in the whole cohort, patients receiving simple vs advanced imaging at hospital arrival had a similar ordinal mRS shift at 90 days (adjusted common OR [cOR] 1.17 [95% CI, 0.96, 1.43]; *P* = .11), good functional outcome at 90 days (adjusted OR 0.83 [0.66, 1.06]; *P* = .13), mortality (adjusted OR 1.20 [0.91, 1.58]; *P* = .20) and sICH (adjusted OR 1.25 [0.61, 2.57]; *P* = .55) (Table 3). However, patients receiving simple imaging had a higher risk of any haemorrhagic transformation than patients receiving advanced imaging (adjusted OR 1.57 [1.00, 2.47]; *P* = .05). Of note, sICH was only evaluated in patients undergoing reperfusion treatment. In the pre-specified sensitivity analysis additionally including baseline site of arterial occlusion and replacing time-to-hospital-arrival tertiles with quartiles (6.7, 10.1, 13.6 h) in the propensity-score model, results were materially unchanged for all outcomes (Table S4)

**Table 3:** Efficacy and safety outcomes in the complete cohort and subgroup analysis. Common odds ratio (for the ordinal mRS) and odds ratio (for mRS 0–2, mortality and sICH) and 95% CI for the adjusted IPW models.

	Ordinal mRS	mRS 0–2	Mortality	Symptomatic ICH
**All (*n* = 1262)**	1.17 [0.96, 1.43], *P* = .11	0.83 [0.66, 1.06], *P* = .13	1.20 [0.91, 1.58], *P* = .20	1.25 [0.61, 2.57], *P* = .55
Subgroups				
** Non-treated (*n* = 716, *n*_sICH_ = 0)**	1.11 [0.86, 1.45], *P* = .42	0.81 [0.56, 1.17], *P* = .26	1.14 [0.77, 1.68], *P* = .51	–
** Treated (*n* = 546, *n*_sICH_ = 522)**	1.28 [0.94, 1.73], *P* = .11	0.85 [0.62, 1.15], *P* = .29	1.28 [0.86, 1.91], *P* = .22	1.37 [0.66, 2.87], *P* = .40
** Primary EVT (*n* = 459, *n*_sICH_ = 283)**	1.46 [1.05, 2.04], *P* = .03	0.70 [0.46, 1.06], *P* = .09	1.40 [0.91, 2.16], *P* = .13	1.79 [0.69, 4.63], *P* = .23
** IVT received (*n* = 87, *n*_sICH_ = 50)**	0.83 [0.39, 1.80], *P* = .64	1.25 [0.53, 2.99], *P* = .60	0.98 [0.33, 2.89], *P* = .96	–
** Distal (*n* = 287, *n*_sICH_ = 133)**	1.05 [0.69, 1.60], *P* = .82	1.14 [0.70, 1.84], *P* = .60	1.43 [0.78, 2.60], *P* = .24	2.82 [0.65, 12.36], *P* = .17
** Proximal (*n* = 451, *n*_sICH_ = 290)**	1.34 [0.95, 1.87], *P* = .09	0.65 [0.41, 1.03], *P* = .07	1.26 [0.84, 1.89], *P* = .25	0.82 [0.33, 2.06], *P* = .68
** No transfer (*n* = 999, *n*_sICH_ = 475)**	1.16 [0.93, 1.45], *P* = .18	0.87 [0.67, 1.14], *P* = .31	1.18 [0.87, 1.61], *P* = .29	1.44 [0.70, 2.99], *P* = .32
** Transferred (*n* = 263, *n*_sICH_ = 47)**	1.20 [0.78, 1.86], *P* = .41	0.71 [0.42, 1.19], *P* = .19	1.26 [0.68, 2.32], *P* = .46	–
** Unknown onset (*n* = 1118, *n*_sICH_ = 287)**	1.14 [0.92, 1.41], *P* = .24	0.87 [0.67, 1.12], *P* = .27	1.16 [0.87, 1.56], *P* = .31	1.70 [0.66, 4.39], *P* = .27
** Known onset (*n* = 167, *n*_sICH_ = 31)**	1.52 [0.87, 2.66], *P* = .14	0.64 [0.34, 1.20], *P* = .17	1.67 [0.66, 4.25], *P* = .28	–

Abbreviations: IPW, inverse probability weighting; IVT, intravenous thrombolysis, *n*_sICH_, sample size for sICH.

Treated includes all patients who received EVT and/or IVT.

### Study of subgroups

**Figure 3:**
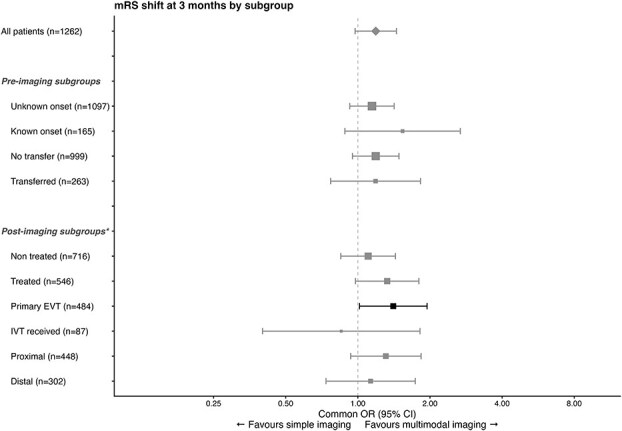
Simple vs advanced neuroimaging for mRS shift at 3 months for each subgroup and for the all the study population. Common OR and 95% CI for the adjusted IPW model. *Pre-imaging subgroups (onset certainty, transfer status) are not affected by the imaging modality assignment. Post-imaging subgroups (treatment received, occlusion site) condition on post-exposure variables and may be subject to collider bias; these results should be interpreted with caution*. Abbreviations: IPW = inverse probability weighting; OR = odds ratio.

Figure 3 shows the subgroup analysis for ordinal mRS and good functional outcome. All subgroups showed a trend towards better functional outcomes with advanced imaging; this trend approached statistical significance in patients with proximal occlusion (Table 3). The only statistically significant result was observed in patients undergoing primary EVT (adjusted cOR 1.46 [1.05–2.04]; *P* = .03); however, this subgroup analysis conditions on a post-exposure variable and should be interpreted with caution. Subgroup analyses stratified by treatment received (EVT, IVT) or by variables determined after imaging (detected arterial occlusion site) condition on post-exposure variables that may act as colliders. Because the imaging modality influences both the detection of arterial occlusion and the subsequent decision to administer reperfusion therapy, stratifying on these variables can induce spurious associations between the exposure and unmeasured patient characteristics within each subgroup. We therefore consider the pre-imaging subgroups (onset certainty, direct admission vs transfer) as the most reliable stratified analyses.

## Discussion

Our study shows that, in patients with moderate-to-severe anterior circulation ischaemic stroke (defined here as NIHSS ≥ 6) admitted between 6 and 24 h after symptom onset, initial evaluation with simple imaging was not associated with a statistically significant difference in 90-day functional outcome compared with advanced imaging. This lack of difference persisted across subgroup analyses, including patients treated or not treated with EVT, those transferred from primary stroke centres, and those directly admitted to a comprehensive stroke centre. Although there was a trend towards better functional outcomes in patients with proximal occlusion who underwent advanced imaging, this did not reach statistical significance.

Regarding safety outcomes, we did not observe differences in 3-month mortality or sICH between the two techniques. However, patients who underwent EVT after simple imaging had higher odds of developing any haemorrhagic transformation compared to those assessed with advanced imaging. Furthermore, we observed a higher percentage of arterial occlusion and a slightly higher rate of EVT in the advanced imaging group, which is in line with previous research.^12^ Unlike previous studies, we found no differences in door-to-puncture times between the two groups. This fact may be explained because, in our setting, interventional teams are on call during nonworking hours and are only dispatched to the hospital when a patient suitable for EVT is admitted. Thus, the team transportation time—more than the imaging acquisition—is probably the main factor delaying EVT in our scenario. Recent observational data from selected academic centres^13^^,^^14^ suggest that the added workflow time of advanced imaging does not translate into worse outcomes. However, these studies compared MRI-first vs CT-first pathways (not CTP vs NCCT + CTA), were subject to feasibility-driven selection and to the ASPECTS artefact between CT and MRI, and their findings may therefore not be directly transposable to a real-world regional network.

Three caveats should frame our findings. First, our exposure is a compound imaging-guided strategy, so the effect reflects a pragmatic pathway (similar to a cluster-randomised protocol comparison), not the imaging technique in isolation. Second, advanced imaging may also aid prognostication; our design only captures its role in reperfusion decisions. Third, the higher “no-occlusion” rate with simple imaging (44.9% vs 37.2%) likely reflects CTP’s greater sensitivity for distal occlusions rather than true differences in pathology.

Previous observational studies have compared stroke prognosis after EVT in late window patients selected by simple or advanced neuroimaging. A meta-analysis gathering evidence from 5 retrospective studies found that both imaging modalities were comparable in functional independence at 90 days and sICH, but patients receiving advanced imaging had a lower mortality rate.^15^ Unlike our study, these analyses excluded untreated patients, introducing a selection bias that limits the ability to determine the most appropriate imaging strategy for guiding treatment decisions in the broader population of patients with suspected stroke. Another study found no differences in clinical outcomes when comparing patients with LVO in the late window arriving at centres that predominantly performed simple imaging against those arriving at centres that predominantly acquired advanced imaging.^16^ Again, only LVO patients were studied, which results in selection bias, as arterial occlusion is only detected after (and not before) imaging evaluation.

Two randomised clinical trials—MR CLEAN-Late and RESILIENT-Extend—have evaluated the safety and efficacy of EVT in the extended time window using simple imaging for patient selection. MR CLEAN-Late demonstrated that EVT is safe and effective under this imaging paradigm; however, its population is not directly comparable to ours, as patients meeting guideline-based criteria for EVT were excluded.^13^ The RESILIENT-Extend trial (ClinicalTrials.gov Identifier: NCT04256096) has shown promising results but its data are still pending peer review and final publication.

Additionally, several studies have reported that EVT improves functional outcomes in stroke patients with large ischaemic cores selected by simple imaging.^17–21^ Nevertheless, expert consensus suggests that the generalisability of these findings to the extended time window remains uncertain, and current recommendations still favour the use of advanced imaging in such cases.^22^^,^^23^ Importantly, none of these studies directly compared simple vs advanced imaging paradigms for treatment selection in the extended window. Our study addresses this gap by evaluating both imaging strategies in a real-world population, including untreated patients, thereby providing a broader perspective on imaging-based decision-making in late-presenting stroke.

In summary, simple imaging was not associated with statistically significantly worse functional outcomes than advanced imaging for guiding treatment decisions in the late window of stroke in a real-world clinical setting. The absence of a statistically significant difference should not be equated with equivalence: point estimates consistently favoured advanced imaging (cOR ~ 1.10–1.46), and the study may be underpowered for modest but clinically meaningful differences. In addition, the richer information provided by advanced imaging—such as the better identification of arterial occlusions due to CTP—should not be overlooked, as it may increase the likelihood of offering reperfusion therapies to eligible patients and reduce the risk of any haemorrhagic transformation, a factor often underestimated but recently recognised as a predictor of poorer prognosis.^24^ Beyond reperfusion selection, advanced imaging may also improve diagnostic performance by helping to identify stroke mimics. CTP can disclose patterns inconsistent with acute ischaemia (eg, absence of any perfusion deficit or focal hyperperfusion suggestive of a seizure-related phenomenon), and thereby reduce the rate of unnecessary reperfusion therapy and procedural complications in non-stroke patients. Our cohort was restricted to confirmed ischaemic stroke and therefore cannot quantify this benefit, but it is an important component of the overall clinical value of advanced imaging that should not be inferred away from our results.

The strengths of our study are (1) a target trial emulation framework that tries to emulate a randomised study, (2) a study population extracted from a prospective mandatory database in which all patients admitted with a stroke in a Catalan centre are registered, decreasing the risk of selection bias, (3) all images were evaluated by a central core lab by experienced neuroradiologists and stroke neurologists from comprehensive stroke centres and (4) mRS was centrally evaluated by a team of qualified nurses unaware of the imaging modality at admission.

Our study also has some limitations. First, this is a retrospective study including stroke patients who received simple or advanced imaging according to the criteria of the neurologist in charge. Thus, we cannot exclude bias in the diagnostic test selection. We used a target trial emulation framework with a pre-specified causal estimand. Because the exposure is a compound strategy, the effect reported is the total effect of the pathway, not the isolated effect of imaging. The validity of this compound exposure rests on the assumption that the strategy was well-defined and reasonably consistently implemented across centres; in a pragmatic, multicentre setting, between-centre differences in workflow, eligibility thresholds and post-imaging decision-making may have introduced heterogeneity that the propensity-score model cannot fully capture. Confounding by indication cannot be fully excluded, and power is limited for low-event outcomes such as sICH. Finally, our results speak only to imaging as a selection tool for reperfusion, not to its value for stroke-mimic exclusion, aetiological work-up or prognostication. In addition, key imaging-derived covariates used to estimate the propensity score—in particular ASPECTS and occlusion status—are known to be subject to inter-reader variability. Inverse probability weighting reweights the distribution of measured baseline covariates between exposure groups under the assumption of correct model specification, but it does not correct for measurement error or misclassification in those covariates. Residual confounding from this source therefore cannot be excluded, and may bias the estimated effect of the imaging-guided strategy. Second, all stroke patients were included in the study, but not all stroke suspicions. Therefore, we do not have information about the performance of the different imaging modalities in identifying stroke mimics. Third, subgroup analyses should be interpreted cautiously: statistical power is limited, and stratifications based on post-imaging variables may introduce collider bias, since these variables are themselves influenced by the imaging modality. Notably, the only statistically significant subgroup result was observed among patients undergoing primary EVT (cOR 1.46 [1.05–2.04]), the subgroup most directly influenced by the imaging modality itself. This finding is therefore particularly susceptible to collider bias and should not be interpreted as evidence that the benefit of advanced imaging is restricted to EVT candidates. Fourth, imaging modalities were not equally distributed among all the stroke centres, which also have different stroke teams and patients regarding their socioeconomic backgrounds. We adjusted our model for the admitting centre to account for these differences.

## Conclusion

In conclusion, in patients with moderate-to-severe anterior circulation stroke admitted within the extended time window, simple imaging was not associated with worse functional outcomes than advanced imaging for guiding stroke treatment. The absence of a statistically significant difference should not be interpreted as evidence of equivalence, as our study was not powered as a formal equivalence trial. However, the richer information provided by advanced imaging may decrease haemorrhagic risk and enhance access to reperfusion therapies in selected patients.

## Supplementary Material

Supplementary_material(1)_aakag068

## Data Availability

The first and the last author (A.V.C. and M.H.P.) had access to all anonymised data. Anonymised data are available upon reasonable request to the corresponding author.

## References

[1] Turc G, Bhogal P, Fischer U, et al. European Stroke Organisation (ESO)-European Society for Minimally Invasive Neurological Therapy (ESMINT) guidelines on mechanical thrombectomy in acute ischaemic stroke endorsed by Stroke Alliance for Europe (SAFE). Eur Stroke J. 2019;4:6–12. 10.1177/2396987319832140 31165090 PMC6533858

[2] Powers WJ, Rabinstein AA, Ackerson T, et al. Guidelines for the early management of patients with acute ischemic stroke: 2019 update to the 2018 guidelines for the early management of acute ischemic stroke: a guideline for healthcare professionals from the American Heart Association/American Stroke Association. Stroke. 2019;50:50. 10.1161/STR.0000000000000211 31662037

[3] Nogueira RG, Jadhav AP, Haussen DC, et al. Thrombectomy 6 to 24 hours after stroke with a mismatch between deficit and infarct. N Engl J Med. 2018;378:11–21. 10.1056/nejmoa1706442 29129157

[4] Albers GW, Marks MP, Kemp S, et al. Thrombectomy for stroke at 6 to 16 hours with selection by perfusion imaging. N Engl J Med. 2018;378:708–718. 10.1056/NEJMoa1713973 29364767 PMC6590673

[5] Nguyen TN, Abdalkader M, Nagel S, et al. Noncontrast computed tomography vs computed tomography perfusion or magnetic resonance imaging selection in late presentation of stroke with large-vessel occlusion. JAMA Neurol. 2022;79:22–31. 10.1001/jamaneurol.2021.4082 34747975 PMC8576630

[6] Dhillon PS, Butt W, Podlasek A, et al. Perfusion imaging for endovascular thrombectomy in acute ischemic stroke is associated with improved functional outcomes in the early and late time windows. Stroke. 2022;53:2770–2778. 10.1161/strokeaha.121.038010 35506384 PMC9389941

[7] Nogueira RG, Haussen DC, Liebeskind D, et al. Stroke imaging selection modality and endovascular therapy outcomes in the early and extended time windows. Stroke. 2021;52:491–497. 10.1161/strokeaha.120.031685 33430634

[8] Hernán MA, Robins JM. Using big data to emulate a target trial when a randomized trial is not available: table 1. Am J Epidemiol. 2016;183:758–764. 10.1093/aje/kwv254 26994063 PMC4832051

[9] Wahlgren N, Ahmed N, Dávalos A, et al. Thrombolysis with alteplase for acute ischaemic stroke in the safe implementation of thrombolysis in stroke-monitoring study (SITS-MOST): an observational study. Lancet. 2007;369:275–282. 10.1016/S0140-6736(07)60149-4 17258667

[10] Souza LCS, Yoo AJ, Chaudhry ZA, et al. Malignant CTA collateral profile is highly specific for large admission DWI infarct core and poor outcome in acute stroke. Am J Neuroradiol. 2012;33:1331–1336. 10.3174/ajnr.A2985 22383238 PMC3888794

[11] Hernán MA, Robins JM. Causal Inference: What If. Chapman & Hall/CRC; 2025: Accessed 13 April 2026. https://miguelhernan.org/whatifbook.

[12] Olive-Gadea M, Requena M, Diaz F, et al. Systematic CT perfusion acquisition in acute stroke increases vascular occlusion detection and thrombectomy rates. J Neurointerv Surg. 2022;14:1270–1273. 10.1136/neurintsurg-2021-018241 34857668

[13] Rapillo CM, Dunet V, Salerno A, et al. Moving from CT-first to MRI-first paradigm in acute ischemic stroke: treatment rates, time metrics, safety, and outcomes. J Stroke. 2025;27:390–401. 10.5853/jos.2025.02229 41084293 PMC12527576

[14] Fladt J, Ospel JM, Singh N, Saver JL, Fisher M, Goyal M. Optimizing patient-centered stroke care and research in the prehospital setting. Stroke. 2023;54:2453–2460. 10.1161/STROKEAHA.123.044169 37548010

[15] Kobeissi H, Ghozy S, Adusumilli G, et al. CT perfusion vs noncontrast CT for late window stroke thrombectomy. Neurology. 2023;100:e2304–e2311. 10.1212/WNL.0000000000207262 36990720 PMC10259276

[16] Lopez-Rivera V, Abdelkhaleq R, Yamal J-M, et al. Impact of initial imaging protocol on likelihood of endovascular stroke therapy. Stroke. 2020;51:3055–3063. 10.1161/STROKEAHA.120.030122 32878563

[17] Costalat V, Jovin TG, Albucher JF, et al. Trial of thrombectomy for stroke with a large infarct of unrestricted size. N Engl J Med. 2024;390:1677–1689. 10.1056/NEJMoa2314063 38718358

[18] White R, Gembreska K, Chaubal V, et al. Thrombectomy for stroke with large infarct on noncontrast CT. JAMA. 2024;332:1355. 10.1001/jama.2024.13933 39374319 PMC11420819

[19] Bendszus M, Fiehler J, Subtil F, et al. Endovascular thrombectomy for acute ischaemic stroke with established large infarct: multicentre, open-label, randomised trial. Lancet. 2023;402:1753–1763. 10.1016/S0140-6736(23)02032-9 37837989

[20] Sarraj A, Hassan AE, Abraham MG, et al. Trial of endovascular thrombectomy for large ischemic strokes. N Engl J Med. 2023;388:1259–1271. 10.1056/NEJMoa2214403 36762865

[21] Huo X, Ma G, Tong X, et al. Trial of endovascular therapy for acute ischemic stroke with large infarct. N Engl J Med. 2023;388:1272–1283. 10.1056/NEJMoa2213379 36762852

[22] Gonzalez NR, Khatri P, Albers GW, et al. Large-core ischemic stroke endovascular treatment: a science advisory from the American Heart Association. Stroke. 2025;56:e87–e97. 10.1161/STR.0000000000000481 39687997

[23] Chen H, Lee JS, Michel P, Yan B, Chaturvedi S. Endovascular stroke thrombectomy for patients with large ischemic core. JAMA Neurol. 2024;81:1085. 10.1001/jamaneurol.2024.2500 39133467

[24] Guasch-Jiménez M, Díaz GE, Lambea-Gil Á, et al. Influence of asymptomatic hemorrhagic transformation after endovascular treatment on stroke outcome: a population-based study. Neurology. 2025;104:e213509. Accessed 17 September 2025. 10.1212/wnl.0000000000213509 40228188

