# Supplementary material for: Simple against advanced imaging for the selection of stroke therapy in the extended window (VESTA study)
Source: Eur Stroke J. 2026 Jun 17;11(6):aakag068. doi: 10.1093/esj/aakag068 (PMC13275002; doi:10.1093/esj/aakag068)

**Supplemental Data**

**Imaging core lab in alphabetical order:**

Lucía Aja (neuroradiologist and neurointerventionist) , Saima Bashir (stroke neurologist and neurointerventionist), Briggitte Beltrán (neuroradiologist), Carlos Castaño (neuroradiologist and neurointerventionist), Marta de Dios (neuroradiologist and neurointerventionist), Ludovica Gramegna (neuroradiologist), María Hernández-Pérez (stroke neurologist, expertise in neuroimaging), Sebastià Remollo (neuroradiologist and neurointerventionist), Isabel Rodríguez-Caamaño (neuroradiologist and neurointerventionist), Mikel Terceño (stroke neurologist and neurointerventionist)

**Supplementary table 1. Target trial inclusion and exclusion criteria**

|  | Hypothetical randomized trial | CICAT registry |
| --- | --- | --- |
| **Inclusion criteria** | Suspicion of anterior acute stroke | Acute stroke suspected anterior |
|  | NIHSS >=6 | NIHSS >=6 |
|  | >18 years, no superior age limit | >18 years, no superior age limit |
|  | Time from symptom onset 6-24h | Time from symptom onset 6-24h |
|  | Simple and advanced imaging available at admission | Admission to centers in which both simple and advanced imaging are available |
| **Exclusion criteria** | Known iodinated contrast allergy | Iodinated contrast allergy not registered |
|  | Modified Rankin Score > 2 | Modified Rankin Score > 2 |
|  | Hemorrhage at NCCT | Hemorrhage at NCCT |

CICAT: Catalan Acute Stroke Registry (Registre Codi Ictus Catalunya). NIHSS: National Institutes of Health Stroke Scale. NCCT: Non-contrast CT

**Supplemental Table 2. Endovascular treatment criteria for each comprehensive stroke center**

| **Stroke center** | **NCCT; ASPECTS limits** | **NCCT; clear hypodensity already established** | **CTA collaterals for selection** | **CTA site of occlusion (including M2, M3 or more distal?)** | **CTP criteria** | **Other criteria** |
| --- | --- | --- | --- | --- | --- | --- |
| **CSPT** | ASPECTS ≥ 6 | No clear hypodensity | No | ICA, M1 and M2 | Core <70 mL and mismatch according to the neurologist | If CTP not available, ASPECTS ≥ 6 and proximal occlusion |
| **HAV** | ASPECTS ≥ 6 | No clear hypodensity | No | ICA, M1 and M2 | Core<70mL, mismatch >1.8 and >15mL | If CTP not available, ASPECTS ≥ 6 and proximal occlusion |
| **HB** | ASPECTS>6 | No clear hypodensity | No | ICA, M1 and proximal M2(with NIH>6) | Core<50 cc and significant mismatch >1.3 (with severe simptomatic oclusion/eloquent area, etc..) | Absence of previous hemorragic tranformation or multivessel occlusion. |
| **HC** | ASPECTS ≥6 | No clear hypodensity | No | ICA, M1 and proximal M2. Also ACA without clear lesion on CT or CBV maps. | ASPECTS>6 on CBV maps. |  |
| **HGTiP** | ASPECTS ≥ 6 | No clear hypodensity | No | ICA, M1 and proximal M2 | Core<70mL, mismatch >1.8 and >15mL | If CTP not available, ASPECTS ≥ 6 and proximal occlusion |
| **HJT** | ASPECTS ≥ 6 | No clear hypodensity | No | ICA, M1 and M2; M3 if eloquent area involvement | Core<70mL and mismatch >1.2 + eloquent area involvement | If CTP not available, ASPECTS ≥ 6 and proximal occlusion |
| **HJXXIII** | ASPECTS>6 on CBV maps | Relative exclusion criteria (clinical/core missmatch) | No | TICA, M1, proximal M2 | Core<70mL, mismatch >1.8 and >15mL (no automated) |  |
| **H MAR** | ASPECTS ≥ 6 | No clear hypodensity | No | ICA, M1 and M2 | Core <70 mL and mismatch according to the neurologist | If CTP not available, ASPECTS ≥ 6 and proximal occlusion |
| **HSP** | ASPECTS ≥ 6 | No clear hypodensity | No | ICA, TICA, M1, M2, and also A1 (if no clear lesion seen according to CBV) | Core<70mL, mismatch >1.8 and >15mL | If CTP not available, ASPECTS ≥ 6 and proximal occlusion |
| **HVH** | ASPECTS ≥ 6 | No clear hypodensity | No | ICA, M1 and M2 | Core<70mL, mismatch not evaluated for taking therapeutic decisions | If CTP not available, ASPECTS ≥ 6 and proximal occlusion. Young patients (<60y), ASPECTS more permissive |

CPST: Corporació Sanitaria Parc Taulí (Sabadell, Barcelona). HAV: Hospital Arnau de Vilanova (Lleida). HB: Hospital de Bellvitge (Hospital de Llobregat, Barcelona). HC: Hospital Clínic i Provincial de Barcelona. HGTiP: Hospital Universitari Germans Trias I Pujol (Badalona, Barcelona). HJT: H. Dr Josep Trueta (Girona). HJXXIII: Hospital Joan XXIII (Tarragona). H Mar: Hospital del Mar (Barcelona). HSP: Hospital de Sant Pau i la Santa Creu. HVH: Hospital de la Vall d’Hebrón

**Supplemental Table 3. Imaging modality according to admission centre**

| **Stroke center** | **Advanced**  N=712 | **Simple**  N=550 | **p.overall** |
| --- | --- | --- | --- |
|  |  |  | <0.001 |
| **CSPT** | 16 (48.5%) | 17 (51.5%) |  |
| **HAV** | 7 (26.9%) | 19 (73.1%) |  |
| **HB** | 187 (50.8%) | 181 (49.2%) |  |
| **HC** | 117 (62.9%) | 69 (37.1%) |  |
| **HGTiP** | 91 (67.4%) | 44 (32.6%) |  |
| **HJT** | 63 (49.2%) | 65 (50.8%) |  |
| **HJXXIII** | 26 (36.1%) | 46 (63.9%) |  |
| **H Mar** | 7 (20.0%) | 28 (80.0%) |  |
| **HVH** | 181 (79.0%) | 48 (21.0%) |  |

CPST: Corporació Sanitaria Parc Taulí (Sabadell, Barcelona). HAV: Hospital Arnau de Vilanova (Lleida). HB: Hospital de Bellvitge (Hospital de Llobregat, Barcelona). HC: Hospital Clínic i Provincial de Barcelona. HGTiP: Hospital Universitari Germans Trias I Pujol (Badalona, Barcelona). HJT: H. Dr Josep Trueta (Girona). HJXXIII: Hospital Joan XXIII (Tarragona). H Mar: Hospital del Mar (Barcelona). HSP: Hospital de Sant Pau i la Santa Creu. HVH: Hospital de la Vall d’Hebrón

**Supplementary table 4.** Sensitivity analysis after adjustment of propensity score

|  | Ordinal mRS | mRS 0-2 | Mortality | Symptomatic ICH |
| --- | --- | --- | --- | --- |
| PS + Site of occlusion | 1.17 [0.96, 1.43], p=0.13 | 0.84 [0.66, 1.07], p=0.15 | 1.17 [0.88, 1.55], p=0.27 | 1.46 [0.58, 3.68], p=0.42 |
| PS + Changing quartiles | 1.17 [0.96, 1.43], p=0.11 | 0.83 [0.66, 1.05], p=0.13 | 1.20 [0.91, 1.58], p=0.20 | 1.52 [0.60, 3.84], p=0.38 |

**Supplemental Figure 1:** Analysis of the balance of covariates after inverse probability weighting adjustment
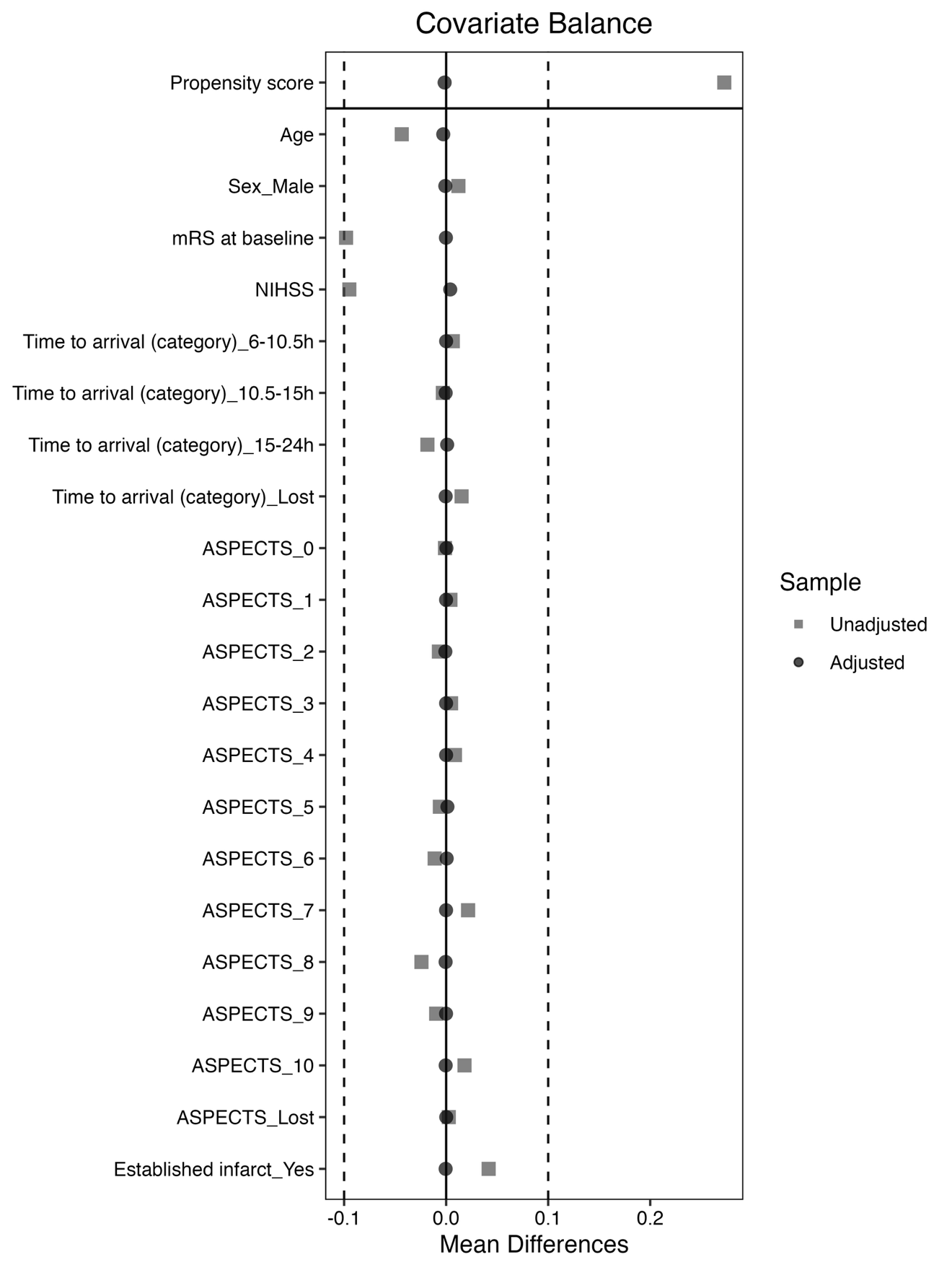

Supplement: Supplementary_material(1)_aakag068 [file supplementary_material(1)_aakag068.docx]
